# The Intrathecal Route for the Administration of Tetanus Antitoxin

**Published:** 1918-01

**Authors:** 


***The Intrathecal Route for the Administration of Tetanus Antitoxin.*** By F. W. Andrewes, Major, R. A. M. C. (T.). Abs. from the *Lancet*, May 5, 1917.

A. argues at considerable length to support the conclusions of the Tetanus Committee, of which he is a member, in favor of the intrathecal route for vaccination against tetanus. He believes that statistics of human cases are far too complicated and meager, at present, to form a basis for any reliable conclusions. Likewise he thinks that theories based upon physiological arguments are practically worthless. Animal experiments, however, may go a long way to prove the efficacy of a route of injection. He cites a considerable body of experiments to prove the value of the intrathecal method, especially those of Permin (Denmark), Park and Nicoll, Golla, and Sherrington. The results of all of these experiments carried out on various animals, were overwhelmingly in favor of the intrathecal route.

A. then gives in detail a group of 20 cases of tetanus that have come under his personnal observation. Of these 16 were treated intrathecally. Only 2 of these died from tetanus. In most cases the recovery was rapid.

The author considers a dose of from 20,000 to 30,000 units in severe cases essential to success by the intrathecal route.

